# 

**Published:** 1901-09

**Authors:** 


Modern Medicine. By Julius L. Salinger, M. D., Demonstrator of Clinical Medicine, Jefferson Medical College, etc., and Frederick J. Kalteyer, M. D., Assistant Demonstrator of Clinical Medicine, Jefferson Medical College, etc. Illustrated; pp. 801; price, cloth, $4.00; half morocco, $5.00. Philadelphia and London: W. B. Saunders & Co. 1900.

This book is not along beaten lines altogether. It presents the practice of internal medicine from the standpoint of a number of specialists, such as physical diagnosis, bacteriology, the examination of the gastric contents, the urine, the blood, the feces, etc. The practitioner of today must be familiar with all of the recent methods for arriving at conclusions in the practice. The man who does not spend some of his time in the laboratory is little less than a prescriber, a mere guesser. This work is particularly prominent in its teachings of the methods of the diagnosis and treatment. Hence the title—Modern Medicine. T. J. B.

